# Darwin's goldmine is still open: variation and selection run the world

**DOI:** 10.3389/fcimb.2012.00106

**Published:** 2012-08-08

**Authors:** Patrick Forterre

**Affiliations:** ^1^Institut PasteurParis, France; ^2^Institut de Génétique et Microbiologie, Université Paris-Sud, CNRS UMR8621Orsay Cedex, France

**Keywords:** evolutionary synthesis, variation, natural selection, lateral gene transfer, Darwinian threshold, viruses

## Abstract

The scientific contribution of Darwin, still agonized in many religious circles, has now been recognized and celebrated by scientists from various disciplines. However, in recent years, several evolutionists have criticized Darwin as outdated, arguing that “Darwinism,” assimilated to the “tree of life,” cannot explain microbial evolution, or else was not operating in early life evolution. These critics either confuse “Darwinism” and old versions of “neo-Darwinism” or misunderstand the role of gene transfers in evolution. The core of Darwin explanation of evolution (variation/selection) remains necessary and sufficient to decipher the history of life. The enormous diversity of mechanisms underlying variations has been successfully interpreted by evolutionists in this framework and has considerably enriched the corpus of evolutionary biology without the necessity to kill the father. However, it remains for evolutionists to acknowledge interactions between cells and viruses (unknown for Darwin) as a major driving force in life evolution.

## Introduction

Darwin had to be defended in the XIX century against those who wished to maintain the concept of our innate highness: the universe had been created for us. The sin of Darwin was to force us to consider ourselves as a “normal” part of the biosphere, and worst, of the animal kingdom. Although Darwin himself, still influenced by biblical thinking, sometimes viewed human beings as the best product of evolution and “natural selection” as a kind of cosmic force (Richards, 2009), his ideas were “dangerous,” because of the powerful explanatory power of the dyad variation plus natural selection and because a descend with modification does not imply progressive evolution (Gould, 1996). Darwin's ideas thus ruined the creationist credo, opening the living world to scientific exploration in the framework of a materialist agenda. Darwin's dangerous idea was defended and he was finally recognized and celebrated in the scientific community. In the last century, the original ideas of Darwin were completed (sometimes corrected) by the development of genetics (the *evolutionary synthesis* or *modern synthesis*) and later on by molecular biology. Recently, Addy Pross proposed a general theory of evolution, extending Darwin's theory to inanimate matter (Pross, 2011). He discusses how Darwin's principles can be deduced from more fundamental chemical principles that govern the evolution of complex chemical systems through imperfect replication and kinetic selection.

Darwin's ideas seem therefore to be alive and well. However, in recent years, whereas still being a devil for religious fanatics, Darwin became the target of heavy criticisms coming from part of the scientific community itself. Several genomists and molecular evolutionists have argued that genomic data have challenged Darwin's view of life (Bapteste et al., 2009; Dagan and Martin, 2009; Doolittle, 2009; Koonin, 2009a,b; Raoult, 2010). Notably, they have suggested that “Darwinism” is only valid for eukaryotes not for prokaryotes (assimilated to microbes) and proposed to replace the “Tree of life” (TOL, supposed to be the hallmark of Darwinism, but see Penny, 2011) by networks (or rhizome) to take into account gene flows between organisms (Bapteste et al., 2009; Dagan and Martin, 2009; Raoult, 2010). In a review title, Ford Doolittle wondered what “*the demise of Charles Darwin*'*s tree of life hypothesis means for classification and the theory of evolution*” (Doolittle, 2009). Lamarck has been (once more) awoken to confront Darwin, as illustrated for instance by the title of another recent review paper: “*Is evolution Darwinian or/and Lamarckian?*” (Koonin and Wolf, 2009).

Carl Woese himself, one of the greatest biologists of the last century, has suggested replacing “Darwininan” evolution (driven by competition between individuals) by communal evolution (driven by exchange of experiences between individuals, via lateral gene transfers) for the early steps of life history, i.e., from the origin of life up to the formation of the three modern cellular domains (Archaea, Bacteria, and Eukarya) (Woese, 2002). He wrote: “*the time has come for biology to go beyond the doctrine of common descent*” and proposed the term “*Darwininan threshold*” to name the transition between communal and “Darwininan” evolution (Woese, 2002). Finally, gradualism and uniformitarianism, considered to be essential pillars of Darwin's view of life, are also (again) strongly attacked (Koonin, 2009a,b).

These views are the bedrock of this special issue with its provocative title “*Microbial genomics challenges Darwin.*” This is a sensitive topic, considering the renewal of creationist thinking in fundamentalist religious circles and the wide publicity given to these “non-orthodox views.” This was best illustrated by the cover of the “*New Scientists*” issue published in January 2009 showing a tree of life superimposed with the sentence: “*Darwin was wrong.*” Although the existence of anti-Darwinists in the political arena is certainly not a reason to hide fierce debates between evolutionists over mechanisms and representations of evolution, one can regret to see the name of Darwin used as a foil in these debates. After all, nobody said: “*Mendel was wrong*” because his concept of the gene was quite different from what we know today (personal quote from Eduardo Rocha).

## Darwin and/or Darwinism

The debate around Darwin and Darwinism is important for the future of our discipline since, as pointed out a few years ago by Bos (1999): “*progress in science is not only a matter of mere technology but of philosophy as well*,” “*progress therefore is reflected in terminology and in the definition of terms.*” In our case, the definition of terms has always been a complex and evolving story. Darwin was not Darwinist. Indeed, although we are biologists, there is no such thing as “biologism.” Scientists are not born to produce doctrines but rational explanations supported by experiments (when possible) and open to criticisms, refutation, and/or modifications. To be consistent with this view, this assay will not be a defense of Darwinism, but of Darwin's core ideas, the couple variation/selection, because Darwinism, as a doctrine, evolved into many different ways, and it is all too easy to select or forge one of them, either to prove it right or wrong. For a recent comprehensive presentation of Darwin's conceptions (beyond the core ideas discussed here), I refer the reader to a recent review by David Penny (2010).

My aim of course is not to argue that Darwin himself was always right, or that we should come back to Darwin's initial views, since, living two centuries ago, he was by necessity ignorant of today biology and he was thinking in another intellectual framework (Richards, 2009). Darwin, originally born Christian and once student in theology, progressively changed his own credo in being confronted to geological and biological facts that could not be explained by the creation theory. Darwin thus finally adopted a materialistic view of the world, putting back human beings into Mother Nature. However, he was still influenced by the *Scala natura* concept of Aristotle (as many modern biologists still are) and his theory initially preserved nature's moral purpose (Richards, 2009). In fact, he thought that “*man is the one great object of nature*” (Darwin, 1987). However, whatsoever Darwin's limitations, I will argue that we have still more to gain standing on his shoulders than tripping him, especially when he cannot reply.

## Selection, yes, but variations first

It is well known that Darwin was not the first to introduce the idea of evolution in biology (beside Lamarck, one of his predecessors was his own grandfather Erasmus) but it's Darwin and Wallace, who were the first to propose a mechanism for the origin of new species: variation followed by natural selection, leading (in Darwin's term) to: “*descend with modifications*,” an expression much more important for Darwin himself than its tree depiction. Although selection does not make sense without variations, “Darwinism” is often reduced to “natural selection” (struggle for life, survival of the fittest) without reference to his emphasis on variation. This is of course because the nature of biological variations remained a complete mystery for Darwin and his contemporaries. In contrast, much was already known on the efficiency of processes such as artificial selection in agriculture and “breeding” and this was determinant for Darwin to formulate his ideas.

Importantly, focusing on natural selection helped the development of evolution as a new branch of biological sciences, the mechanism of natural selection being open to experiments *in situ* (in the fields) as well as in the laboratories, so that evolution became part of the mainstream biological research agenda. From the focus on selection emerged terms such as fitness, genetic drift, the introduction of statistics to “measure” evolution (making it a “true” science) and the creation of new disciplines, such as population genetics.

However, the contribution of Darwin cannot be rightly summarized by natural selection. Darwin also realized the importance of variations, as a prerequisite for evolution. The chapter 2 in “*On the origin of species*” entirely deals with variation, discussing varieties and sub varieties within species, whereas natural selection is discussed in chapters 3 and 4. This is not trivial. At the time of Darwin, biologists were still strongly influenced by current philosophical theories that focus on the essence of things (reminiscent of Plato's ideas). When considering a particular “species,” zoologists or botanists were not fascinated, but rather annoyed, by the diversity of the individual members of this specie. They were looking for the ideal “type species” to describe species without having to mention all possible varieties. For religious scientists, they probably hoped in this way to reconstruct the first member of this species, the one directly created by god (varieties being less perfect by-products).

The great merit of Darwin was to change this perspective upside down. Instead to be confused by the diversity within species, he realized that this diversity is the essence of life, variations providing substrates for selection. As pointed out by Brüssow (2009) “*diversity is not an evolutionary accident, but the organizing principle in biology, without which evolution would not occur.*” The four years of Darwin exploration with the Beagle, far from academic life, were certainly critical in opening his eyes on this issue. In fact, there is no such thing as a species in the real world, except as concepts in our mind (and in books) but organisms and populations. There are myriads of individuals that are all different, even between members of the same “species” defined by any criteria. Darwin was the first to realize that this diversity was the key parameter allowing selection.

The historical focus of most evolutionists on selection, instead of variations, produces some confusion on the nature of selection. Darwin himself used to think of selection somehow as a kind of metaphysical force (Richards, 2009), and similarly, some evolutionists used to consider natural selection as the cause of evolution. As a consequence, each time a new mechanism of variation or any constraint in the mode of existence of organisms is discovered, it is claimed that natural selection has been weakened (see for instance Table 1 in Pigliucci, 2009 in which natural selection is supposed to be *altered* or its *efficacy decreased* by phenomena such as *contingency, biological emergence or phenotypic plasticity in macroevolution*). However, in my opinion, this is quite misleading. Natural selection is not an “evolutionary force” but the necessary outcome of variation and multiplication. In particular, natural selection cannot be weakened by mechanisms that promote variations (such as epigenetic mechanisms or symbiogenesis), because these processes provide more substrates for selection.

Importantly, Darwin realized that natural selection is the inevitable consequence of the extraordinary multiplication power of living organisms. In that sense, microbial evolution does not challenge but vindicates Darwin, since the multiplication power of life is higher by several orders of magnitude in the microbial (and viral) world, making natural selection even more drastic in these realms. Despite the limited knowledge of his time, Darwin himself was in fact the first to consider that microbial evolution also involves natural selection (O'Malley, 2009).

Originally, Darwin mainly (but not exclusively) used to consider what we call now positive selection, we known today that variants can be also selected by chance (genetic drift) or strongly counter selected if they do not fit the basic life requirement of the organism (purifying or negative selection). This has been clearly observed by molecular biologists at the sequence level, as in the case of neutral evolution (Kimura, 1977). It is important to insist once more that any type of selection only makes sense because of variation. If conditions change, the successful variants will not be the same, but in any case, selection will operate as soon as variation exists.

## The nature of variations

The chapter five of “*On the origin of species*” is entirely devoted to the nature of variation (a tour de force, considering the state of the art in biology at his time). In contrast to a widely held assumption, Darwin did not think that variations occurred mainly by random processes (although he recognizes the existence of random variation). He was not opposed to the “inheritance of acquired characters” and agreed with the idea that “use or disuse” of a character led to its progressive gain (fixation) or loss. Two notions that are today associated to “Lamarckism” (see below). His most original idea was that important variations were slight changes induced (mysteriously) by the environment in the reproduction apparatus. Darwin was therefore “Lamarckian,” although his focus on the reproduction apparatus can be interpreted as a premonition of the distinction made later on by Weissman between the soma and the germen. The traditional opposition between “Darwinism” and “Lamarckism,” based on the idea that Darwin would favor random variations whereas Lamarck favored the inheritance of acquired characters is clearly wrong. The major difference between Lamarck and Darwin is that, for Lamarck, evolution came from concerted modifications triggered by an internal “vitalistic” forces (*le pouvoir de vie*), so that all individuals in the population experience similar changes to become more adapted to their environment. In that case, natural selection has no more *raison d'être*. For Darwin, acquired characters (even if triggered by the environment) were, first of all, individual acquisitions that should have survived the screen of selection.

The identification of DNA as the carrier of genetic information in the middle of the last century was a decisive blow for neo-Lamarckism. It was difficult to imagine how environmental changes could modify on purpose the sequence of DNA. Early molecular biologists assimilated variations to random mutations and assumed that environmental modifications cannot produce oriented-mutations. For them, selection (instead of variation) became the *Deus ex machina* who sorts out from the chaos of random mutations those making sense for the organism. The book “*Chance and Necessity*” by Jacques Monod perfectly illustrates the best achievement of this thinking (Monod, 1971). In this book, the dyad variation/selection is replaced by the dyad “chance/necessity” which is supposed to be more or less equivalent. It emphasizes that variations were assumed to be entirely the result of random processes, whereas the result of selection provides THE supposedly unique answer “necessary” to make the organism efficient in a given context.

We know today that molecular mechanisms of variations are much more diverse and complex than simple random punctual mutations. Molecular biologists have expended our concept of variations by revealing the importance of epigenetic systems, whereas cellular biologists have continues to reveal the importance of symbioses as a major form of variation. We also know that many answers are possible for a given situation, providing a much more complex history for life, introducing chance in the process (contingency). But none of these considerations challenge Darwin himself, even if they challenge successive historically dated versions of “Darwinism.”

The great achievement of molecular biology has been to answer (still partially) to one of the most important question in biology: what are the mechanisms of variations? All discoveries of molecular biologists have vindicated Darwin by revealing the molecular mechanisms behind the multiplication and variations of living organisms. With genetic engineering, molecular biologists have finally got the possibility to produce by themselves artificial variations in the genetic material of organisms, making evolution a fully experimental discipline.

## The false come back of Lamarck

Molecular biologists are now out of fashion and spotlights focus on genomists and synthetic biologists. Possibly because Darwin's contribution was clearly recognized by the pioneers of molecular biology (now often accused of reductionism) it seems that genomists and some modern evolutionists look for another hero apparently fitting better with “holistic views” and “systemic biology.” Lamarck (more precisely neo-Lamarckism) is again recruited in this crusade. Recently, when a novel mechanism of genome variation apparently triggered by the environment is discovered, it is often claimed that Lamarck was right and Darwin wrong. For example, it has been recently argued that the discovery of the clustered regularly interspaced short palindromic repeats (CRISPR) system in Bacteria and Archaea is Lamarckian, because these microbes can acquire in their genomes viral sequences that immunize them against future viral infections (Koonin and Wolf, 2009). This is interpreted as a hereditary trait acquired from an environmental modification (the presence of a virus) and oriented by this modification (providing future resistance to THIS particular virus). The inserted viral sequence in the CRISPR locus will be maintained by positive selection if the virus is present (use) but will be lost after some time if the virus is no more encountered (disuses).

However, this interpretation is misleading. Indeed, the addition of new sequences to CRISPR loci would have been considered simply as another form of variation by Darwin. The CRISPR system itself emerged and has evolved through random variations and selection and still works that way. When a bacterial population encounters a particular virus, only part of the population is infected (randomly selected among non-lysogenic bacteria with the proper receptor for this virus). Most infected bacteria are killed, whereas some of them (again randomly selected) survive the infection either because of point mutations affecting host virus interactions or because they have successfully activated a CRISPR defense system. At the end, only a handful of survivors would have acquired new CRISPR sequences from the virus. Finally, these new sequences remain present in the genomes of the survivors only if these descendants are selected (*versus* those losing these sequences) by the continuous presence of this particular virus in the environment. The Lamarckian component of the CRISP system appears dependent of our subjectivity. We forget all selection steps that have modeled random variations into a mechanism that seems to have a purpose because it corresponds to an adaptation of Bacteria (or Achaea) to their environment (the presence of viruses).

It is also often argued that horizontal gene transfers (HGT) are Lamarckian because transferred genes are provided by the environment (contact with another organisms) and lead to a better adaptation to this environment (Koonin and Wolf, 2009). However, evolution is not working that way. Let's consider a schematic scenario (too simplistic but just for the demonstration) of adaptation to different temperatures. Our scenario starts with a species of thermophilic organisms living in a hot environment (70°C) whose temperature corresponds to their optimal growth temperature (OGT). In that environment, these thermophiles should coexist with hyperthermophilic (OGT of 80°C) and moderate thermophiles (OGT of 60°C), because their growth curves in function of temperature would overlap. As a consequence, some thermophiles can gain randomly by HGT either advantageous features to live at lower temperature from moderate thermophiles, or advantageous features to live at higher temperature from hyperthermophiles. If the temperature of the environment changes, different members of the thermophilic species will be selected, depending if the climate is cooling or warming. There is nothing “Lamarckian” in this sketch, but it can be interpreted *a posteriori* as such, because it gives the false impression that HGT have facilitated *a priori* the adaptation of former thermophiles to their new biotope. In fact, HGT are no more “Lamarckian” than “Darwinian.” For a given organism (either a virus or a cell) any type of HGT is simply a particular type of variation (Forterre, 2011b). The same can be said from the acquisition of an organelle via endosymbiosis, even if long term changes introduced by this particular variation can be tremendous (Maynard-Smith, 1991). On a theoretical ground, this type of variation does not differ from a single point mutation. Darwin would have been delighted to learn of HGT and endosymbiosis as powerful tricks for variation providing adaptation to the environment.

## Darwin and the tree of life

In recent years, Darwinism has often been associated to the Tree of life (amazingly written in capital letters by its detractors, TOL). For that reason, I will use the abbreviation Tol thereafter (also to prevent any post-biblical interpretation, see Penny, 2011). However, the supposed love affair between Darwin and trees is restricted to the single figure of his book in “*On the origin of species.*” In fact, Darwin preferred the coral metaphor, in order to emphasize the existence of extinct lineages (unsuccessful variants). For Darwin, the usefulness of the tree metaphor was to illustrate the concept of descend with modification. Each node in his tree exhibits multiple small branches symbolizing variations, one of them producing further bifurcations, all others being dead-ends, symbolizing variations that were counter-selected in the evolutionary game. However, all evolutionists are aware that the actual connections are more complex than those depicted by such simple schematic tree. Some lineages can fuse (sexual eukaryotes in fact evolve through successive fusions of individuals) and several robust branches can emerge from a single node. In fact, the same occurs in actual trees in natural forests (with lianas connecting different branches of the same or different trees). The tree metaphor is thus quite good if one refers to real trees and not simplified versions, although simplified versions are still useful to depict graphically the process of evolution on paper or on computer screens!

It is often claimed that HGT fundamentally contradicts the tree concept (Dagan and Martin, 2006; Doolittle and Bapteste, 2007; Doolittle, 2009; Koonin, 2009a,b). Several authors thus have suggested to replace trees by webs (and TOL by WOL!), in which all genes/organisms are connected by links forming networks in the three dimensional space (Halary et al., 2010). It is also argued that organisms cannot be placed on a tree because they are essentially chimera, produced by fusion of different evolutionary lineages, much like a rhizome (Raoult, 2010). However, as already discussed by several authors, these views confuse species and gene (or genome) trees (Galtier and Daubin, 2008; Gribaldo and Brochier, 2009; Valas and Bourne, 2010). For instance Koonin correctly noticed in his paper entitled “*Darwininan evolution in the light of genomics*” that: *The genomes of all life forms are collections of genes with diverse evolutionary histories.*” Then he conclude surprisingly that: “*a corollary of this generalization is that the TOL concept must be substantially revised or abandoned because a single tree topology or even congruent topologies of trees for several highly conserved genes cannot possibly represent the history of all or even the majority of the genes*” (Koonin, 2009a,b). This clearly demonstrates that in this conclusion, Koonin assimilates the TOL to a tree of genes (or genomes). But for most evolutionists, any “trees of life,” including the Tol should be trees of organisms (either mono or pluricellular), depicting the history of their relationships, from cell to cell or from individual to individual. The histories of genes, genomes and replicons are fascinating (especially for genomists and molecular biologists) but they only make sense if we have access to the history of organisms (otherwise, this would mean that we reduce living organisms to their genomes). Fortunately, the history of any gene, with its duplication and HGT, is constrained by the history of organisms, explaining why accurate analyses of gene trees can sometimes reconstitute efficiently trees of organisms.

The cover of the New Scientist, with a tree superimposed by the sentence, “Darwin was wrong,” has perfectly illustrated the violence of the attack against Darwin, based on the critic of the tree metaphor. For instance, Dagan and Martin (2006) derided the Tol based on universally conserved proteins as the tree of 1%, because it is based on the analysis of about 1% of genes (around 100) in average bacterial or archaeal genomes (Dagan and Martin, 2006). On the contrary, the fact that this tree, reconstructed from so few data, confirmed the tripartite division of cellular life, originally deduced from rRNA sequences comparison (the tree of a single gene, 0.01%!), is for me a triumph of reductionism in studying the history of life. It illustrates the power of comparative sequence analysis (and “tree-thinking”) to reconstruct ancient history (despite all well-known difficulties in resolving ancient nodes, see discussion in Forterre and Philippe, 1999; Forterre, 2010b; Gribaldo et al., 2010).

Interestingly, using holistic approaches, Koonin and colleagues also confirmed the tree of 0.01%, since they conclude that a tree-like signal confirming the tripartite division of life can be recovered from the “*phylogenetic forest*” of gene trees (Puigbò et al., 2009, 2010). Many authors have indeed noticed that phylogenies based on universal proteins and those based on whole genome trees produced more or less congruent global history of life on our planet (at least recovering the three domains). This observation suggests in particular that HGTs have not been so extensive between domains (Wolf et al., 2002; Ciccarelli et al., 2006; Puigbò et al., 2009, 2010). HGT have been indeed extremely rare between domains and between lineages of the same domain for informational proteins such as ribosomal proteins or RNA polymerase subunits (for a case study, see Brochier et al., 2005). Careful phylogenetic analyses of these proteins produce well resolved trees of the archaeal domain that most likely reflect quite accurately the history of this lineage (Brochier et al., 2005; Brochier-Armanet et al., 2011). This means that tentative trees of organisms can be indeed recovered from the forest of gene trees.

**Figure 1:**
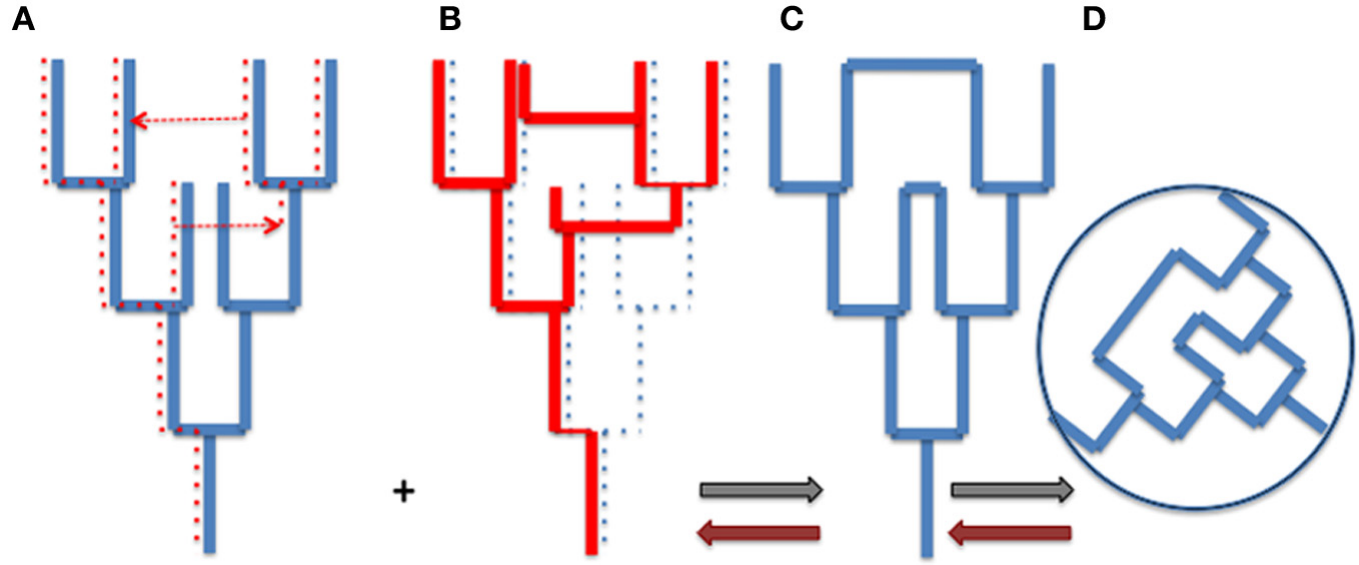
**From trees to webs and back. (A)** an organism tree corresponding to its vertically inherited genes (blue), **(B)** an underlying gene tree [dotted lines in **(A)**] with one loss and two horizontal gene transfers [indicated by dotted arrows in **(A)**], **(C)** the network obtained by combining **(A)** and **(B)** (gray arrows), **(D)** the web corresponding to the unrooted network. Brown arrows indicate the path unveiling the organism and gene trees from the web.

The tree metaphor is not only valid for organisms, but it also works for cells, genes and genomes (or more precisely replicons). In other word, the “*net component of prokaryotic evolution*” (Puigbò et al., 2010) is also tree-like. As soon as an object divides by duplication, the history of that object has a tree-like structure. However, there is no reason why trees of organisms and trees of genes, genomes or replicons should be congruent. The Figure 1 compares a tree of organism (A) and the underlying tree of a particular gene (with one loss and two HGT) (B). Combining the organism and gene trees produces a network (C and D). Importantly, the tree-like structures depicting the history of the organism (A) and the history of the gene (B) is not changed by the HGT (see also Poole, 2009). The structures of organismal trees are not even changed by more drastic variations such as endosymbiosis. The acquisition and enslaving of a cyanobacterium by ancestors of modern plants has not changed the tree-like structure of the eukaryotic domain, viridiplantae emerged as one branch within the eukaryotic tree. Similarly, the endosymbiosis of mitochondria has not changed the tree-like structure of the eukaryotic lineage, defined by the continuity of the cell membrane of the engulfing species (either an archaeon or a proto-eukaryote, depending of your favorite hypothesis, see Gribaldo et al., 2010).

Trees of organisms, genes, and replicons can be of course much more complicated than those depicted by Figure 1, to accomodate hybridation or symbiosis (especially in the case of eukaryotic species), or splitting, fusion, or recombination in the case of genes and replicons. These complications make difficult to represent all evolutionary processes by simple tree-like diagrams, except if one focuses on a particular type of biological or molecular entity, but they can always be interpreted as combination of trees. However, combining all organismal, genomic and replicon trees to get an exhaustive view of life history would produce a monstrous network (the WOL!) that would make sense only if we are able to deconstruct this network to identify the underlying trees and their evolutionary relationships (brown arrows in Figure 1).

At smaller scale, a useful example can be provided by the situation of an amoeba hosting endosymbiotic bacteria, which is infected by a mamavirus, itself infected by a sputnik (satellite virus) (Forterre, 2011b). In that case, we have four organisms living in the same cell. These organisms exchange genes and the cell can be viewed as a melting pot of different organisms (a holobiont). However, each organism maintains its individuality during evolution, the Amoeba remains a eukaryote, the bacterium a bacterium, and the two viruses remain viruses. We have four distinct evolutionary lineages (four putative trees) that need to be sorted out from each other to reflect the reality of organismal evolution. The co-evolution of these organisms in the same cells will of course provide the possibility of more variations induced by interactions between these organisms (such as HGT) and these variations will be selected both at the level of individual organisms and at the level of integrated cell.

Doolittle would possibly argue that views defended here are watered down versions of “Darwinism” (Doolittle, 2009). He suggests indeed that Darwin used fact tree metaphor as a major hypothesis to explain “*the hierarchical structure of tree-like classification.*” In such tree like classification, “*the characters which naturalists consider as showing true affinity between two or more species are those which have been inherited from a common parent.*” According to Doolittle, this hypothesis is incompatible with HGT. It is true that homologous characters common to two or more species may have been acquired by HGT (from an ancestral common parent anyway!) confusing the structure of phylogenetic classification if they are not recognized as such. But we also know from Henning that characters should not be used for natural classification based on the evolutionary history if they are not true synapomorphies, because they do not reflect true affinity (Hennig, 1966). The question is to identify HGT to purge phylogenomic data from sequences that were not vertically inherited and/or to use them as synapomorphies to confirm or identify monophyletic groups (Brochier et al., 2005; Huang and Gogarten, 2006).

## The web-like structure of microbial evolution: an old and wrong idea in new clothes

One of the greatest achievements of biology in the last century has been the inclusion of microbes in the tree of life, thanks to the rRNA tree and the dramatic discoveries of biochemists and molecular biologists, using *Escherichia coli* and its viruses as model systems. For a long time, it was unclear, both for microbiologists and for evolutionists working on animals and plants, if microbes and macrobes (organisms visible by naked eyes, see Forterre, 2008) could be unified into a single tree (although it was already clear for Darwin that microbes were subjected to variation and selection, see O'Malley, 2009). The discovery, in the middle of the last century, that bacteria and their viruses share with the rest of the biosphere the same macromolecules, the same genetic material, and the same genetic code, was a major breakthrough. However, it was not immediately obvious if microbial evolution could be studied in a meaningful way. The existence of HGT through transformation, transduction, or conjugation was recognized early on by pioneers of molecular biology (well before genomists) and it was widely believed that bacteria should be able to share their genes to such an extend that it was pointless to try reconstructing microbial evolution (for an historical account, see Sapp, 2005). Some authors even argued that bacterial “species” should be considered as “cell types” of a gigantic bacterial organism covering the planet (Sonea, 1971; Sonea and Paniset, 1976).

In one of their two seminal 1977 papers, Woese and co-workers remained us that “*the scattered classification”* of methanogenic bacteria *in the seventh edition of Bergey's Manual* (some being Gram positive, other Gram negative bacteria, some bacilliform, others coccoidal or filamentous) was *“rationalized in terms of reticulate evolution involving an appropriate plasmids”* (i.e., HGT of genes involved in methanogenesis) (Fox et al., 1977). Woese and co-workers however demonstrated experimentally, using the tools of molecular biology, that (1) methanogens form a coherent group of organisms very distant to other prokaryotes known at that time, and (2) they are not Bacteria but Archaea (formerly Archaebacteria) (Woese and Fox, 1977). These landmark papers opened the door to a comprehensive history of microorganisms. More recently, phylogenetic analyses have shown that genes involved in methanogenesis, although “operational,” have not even been transferred between different archaea (Bapteste et al., 2005). These analyses have unveiled a complex history, with a single origin for methanogenesis, but several subsequent losses, leading to the formation of two paraphyletic groups of methanogens.

The example of methanogens again shows that it is possible to get meaningful information about the early history of life in our planet, thanks to tree thinking. By refuting the validity of this approach for studying microbial evolution, scientists who called themselves “microbalists” and derided those who refuse to criticize Darwin as “positivists” (Dagan and Martin, 2009) propose in fact to bring us back to the pre-Woesian era, when studying the history of microorganisms was considered to be a futile exercise. Let me consider that the recent proposal of a third major archaeal phylum, the Thaumarchaeota, by “positivist” is another positive outcome of “tree thinking” (Brochier-Armanet et al., 2008). This proposal, now accepted by the community of microbiologists (Spang et al., 2010) has opened new avenues in focusing the attention of evolutionists on this fascinating archaeal phylum. The discovery in this phylum of eukaryotic traits previously unknown in other archaea has raised new questions on their relationship with eukaryotes (Brochier-Armanet et al., 2011, 2012). Such questions can be only addressed meaningfully in a “tree-thinking” framework; they would vanish unfortunately in “web thinking.”

Finally, recent post-genomic works on archaeal or bacterial speciation have emphasized that microbial speciation occur by mechanisms very similar to those occurring in macrobes, despite differences in HGT and sex (Cadillo-Quiroz et al., 2012; Shapiro et al., 2012). Amazingly, whereas Shapiro and co-workers illustrate their experimental work by a simple bifurcation tree showing the divergence of two populations (with progressive reduction of HGT between them), Papke and Gogarten (2012), in an accompanying comment, illustrate the same story by a reticulate diagram (Web thinking) to emphasize these HGT. Surprisingly, they spoke of a “*startling anti-Darwninan outcome*” because extensive gene flow within the original population prevents to define a unique common ancestor containing all genes present in the two diverging populations! However, Darwin, who ignored the existence of genes, never discussed the genetic composition of ancestors of two populations. One again, there is here a clear confusion between genes and organisms.

## The Darwinian threshold and the veil of communal evolution

In the post-genomic era, positivists themselves are not immune to the influence of microbialists. Hence, whereas Woese's brilliant work has experimentally demonstrated that it was possible to decipher the history of microorganisms despite HGT, he reinstated himself later on HGT at the center of the universal tree, hiding the root itself under the veil of communal evolution (Woese, 2000, 2002). Carl Woese introduced the term “*Darwinian threshold*” to characterize the transition between the first period in life history, during which evolution has supposedly occurred mainly through HGT, and the second epoch (we are still living in) characterized by “Darwinian evolution” (formation and diversification of species) (Woese, 2002).

In the communal scenario proposed by Woese, primitive organisms (progenotes) living before the Darwinian threshold exchanged genes (thus characters) freely (and extensively) because they exhibited a loose modular fabric. As a consequence, different genetically encoded modules could be exchanged without damage for the organisms. Innovations occurred nearly simultaneously in the whole biosphere, without the possibility to form stable evolutionary lineages (species). Progenotes could not really compete and be selected since they were all quite similar at all stages of early evolution. Three distinct types of molecular biology finally “crystallized” to form the ancestor of each modern domain (the beginning of speciation) reducing dramatically the possibility of HGT between domains (at least for basic cellular informational processes). In a paper exploring the importance of HGT in the evolution of the genetic code toward universality and optimality, Woese and co-workers wrote that: “*evolution of the genetic code; translation, and cellular organization itself follows a dynamic whose mode is, if anything, Lamarckian*” Vetsigian et al., 2006).

Notably, the question of the root of the universal tree (a fundamental problem in biology) becomes futile in the communal scenario. Carl Woese wrote “*the universal tree has no root in the classical sense, the root is actually a Darwinian threshold*” (Woese, 2002). He simply suggested that Bacteria crossed first the Darwinian threshold, followed by Archaea and Eukarya (in that order) to fit with the traditional rooting in the bacterial branch (Woese, 2002) (ladder thinking, see Forterre, 2010b). A fundamental scientific question is thus abandoned (as in the case of fusion scenarios that roots *a priori* the tree between Archaea and Bacteria). We should renounce, for instance, to determine if traits shared by two of the three domains are ancestral or derived. In fact, we cannot anymore apply cladistic principles of evolutionary phylogenetic (Hennig, 1966) that have been so successful in deciphering the history of macrobes.

In fact, the concept of Darwinian threshold supposes that we abandon the powerful evolutionary mechanism discovered by Darwin (variations plus selection) as an explanation for the most difficult problem of all, how life originated and evolved from inanimate matter to the modern biosphere? This idea is at odd with the principle of continuity, a powerful weapon against creationism, as important now as it was at the time of Darwin (see below). In fact, in the communal scenario, we watered down the extraordinary multiplicative power of life (disruptive of communities), a phenomenon crucial for natural selection, to introduce instead a mysterious progressive force, reminiscent of the *pouvoir de vie* of Lamarck. Hence, Carl Woese wrote: “*a stage inevitably will be reached* (during communal evolution) *were some cellular entities become complex enough that their cell design start to be unique*” (Woese, 2000). This suggests indeed that life evolves inevitably toward complexity, but we do not know how and why.

The communal scenario also supposes that life originated and evolved up to the last common ancestors of the three domains in a very limited spatial environment to allow all cells in the evolving communal population to acquire rapidly any beneficial novelty by HGT. Koonin and Martin (2005) have indeed propose a scenario in which life evolved up to the emergence of Archaea and Bacteria in the confined environment of a single hydrothermal chimney. I think that such scenario is very unlikely. My guess is that (again considering the extraordinary multiplicative power of life) the whole planet was already covered by a biosphere at the time of the Last Universal Common Ancestor (LUCA) of modern cells. LUCA and its contemporaries were indeed already quite complex cellular organisms (Forterre, 2010b) with an already optimized genetic code (Vetsigian et al., 2006). However, there is no experimental data showing that extensive HGT would prevent speciation, even in a confined space. On the contrary, a recent study of archaeal populations living in a single hot terrestrial pool have shown that, even in such confined environment, microbial populations can diverge and form new species despite extensive HGT by acquiring specific traits that allow them to adapt to specific metabolic niches (Cadillo-Quiroz et al., 2012). The same should have occurred for ancient (pre-LUCA) microbial populations, except if we assume that no ecological differentiation take place at the time of LUCA, something very unlikely.

Fortunately, there is no reason to abandon Darwin's vision when we think of primordial evolution, even if HGTs were more prevalent during this period of life history (something *a priori* not obvious since HGT involves presently complex molecular mechanisms for DNA transfer from one cell to another). As previously mentioned, HGT cannot *per se* modify the nature of the evolutionary process (Forterre, 2012). Novelties can be transmitted by HGT but only fixed via the selection of individual. One could argue that HGT were so frequent before LUCA that a beneficial gene was rapidly transferred to the whole population, but the same would have been true for deleterious HGT! Parasites would have also invaded and disrupted such communal population (Poole, 2009). Again, only selection between a myriad of individuals could have make sense of these variations.

Positive selection was certainly at work, as a result of competition between parasites, predators, and preys (Forterre, 2005). Beside, neutral and purifying selection can have also play a major role in building cellular complexity (Lukeš et al., 2011). In all cases, variation and selection were certainly the two pillars of life evolution in the pre-LUCA era as they are now. The formation of stable species might have indeed been prevented, but Darwinism cannot be assimilated to the formation of stable species, especially since, as previously discussed, the great merit of Darwin had been to focus on the variability of organisms and the lack of natural barrier between species, varieties and sub-varieties.

Even in the absence of “speciation,” evolution of individual living entities before LUCA should be viewed in a tree-thinking framework. The evolution of replicators, protocells, and RNA-based cells produced myriads of trees in our distant past, and the best (or the lucky) replicators, protocells, and RNA-based cells were selected at each turning point of life history (the first organism with genomic RNA distinct from ribozyme RNA, the first organism with ribosomes producing encoded peptides, and the first organism with the modern genetic code, the first organism with DNA genomes). The winners were selected whereas many other types of organisms that once existed were eliminated (for instance organisms producing proteins—with possibly D amino-acids—using RNA-based machineries distinct from the ancestors of the ribosome). These selections were meaningful, thanks to variations that occurred in RNA replicons. Back in time, these complex chemical processes based on variation and selection probably originated from chemical kinetics selection that can favor the emergence of more complex replicators through imperfect replication (variation) and kinetic selection (Pross, 2011).

Why did Carl Woese propose the concepts of Darwininan threshold and communal evolution? One of his aims was probably to explain why the tempo of evolution was much higher at the time of LUCA (and before) than it became later on recently (Woese, 1998 and references therein). More precisely, he wonders why three different versions of universal proteins emerged in the relatively short period between the origin of proteins and LUCA, whereas they remained relatively similar within each domain until now, i.e., during a much longer period? This is an important question that Carl Woese raised early on, promoting the progenote concept (weak coupling between genotypes and phenotypes) as one possible explanation for the difference in evolutionary tempo before and after LUCA (Woese, 1998 and references therein). By introducing the Darwininan threshold concept, Woese now suggests that the tempo of evolution was faster before this threshold because the spread of characters by HGT accelerated the path of evolution. In his new model, three different versions of universal protein were established because the three domains crossed the “Darwininan threshold” at different times.

Explaining the change in evolutionary tempo that take place after the formation of the three domains is indeed an important issue. Hervé Phillipe and myself talked of: “*three dramatic evolutionary events*” that reduced this tempo at the onset of the three domains (Forterre and Philippe, 1999). I once suggested that these events were coupled to three independent transitions from RNA to DNA genomes that reduced the rate of genome evolution at the origin of each domain (Forterre, 2006a). Other hypotheses can be certainly put forward. However, I do not think that such events could be simply explained by a decrease in HGT frequency. In fact, we have no idea of the variation of HGT frequency during the history of life. Considering that HGT require today complex molecular machines for DNA transfer, it might be even possible that HGT were less frequent in ancient time than they are now. In fact, one could for instance argue that HGT were very rare at the time of LUCA, explaining why the three domains could indeed diverged so much, whereas more extensive HGT later on within domains homogenized molecular biology within but not between domains.

## Uniformitarianism and gradualism

Darwin has insisted on gradualism (*natura non facit saltum*) because he had to fight against creation theories. He promoted the idea that evolution proceeds via the gradual accumulation of tiny modifications. This view is now another angle of attack against “Darwinism.” In particular, genomics would have teached us that “*Natura facit saltum*” (see below) and that Darwin was wrong to insist on gradualism and uniformitarism in the biosphere (Koonin, 2009a,b). However, the history of science tells us that it does not make sense to claim that Darwin was wrong each time we discover a variation that is not so “gradual” after all. At the beginning of the XX century, “mutationism” was opposed to “Darwinism” because mutations were discrete, not gradual, modifications (a red eye becoming white) until population geneticists finally reconciled mutationism and Darwinism, showing that “*multiple Mendelian factors combined with random environmental effect to give apparently continuous variations*” (Barton et al., 2007). Today, it is claimed that HGT (once more), endosymbiosis, gene or genome duplications are not gradual too, introducing discontinuities in life history. For instance, Koonin (2009a,b) noticed that “*genome duplication is far from being an infinitesimal change.*” However, Darwin, who has no idea of the nature of variation, was speaking of « tiny modifications » at the phenotypic level. This debate in some way brings us back to the pre-modern synthesis era, instead of opening post-modernist avenues! Once again, if we look closely at the evolutionary process, I do not think that we need to forget gradualism so easily.

Let's take one example, mitochondrial endosymbiosis, *a priori* a dramatic discontinuity in the history of eukaryotes (and often viewed as such). The story started with a proto-eukaryote engulfing an alpha-proteobacterium. Interestingly, some modern eukaryotic cells still harbor endosymbiotic alpha-proteobacteria (Beninati et al., 2004; Park et al., 2009). Amazingly, in the tick *Ixodes ricinus*, the alpha-proteobacterium endosymbionts even parasitize mitochondria themselves (Beninati et al., 2004). The infected eukaryotic cells are not visibly different from their close relatives lacking endosymbionts. Similarly, the entrance of the alpha proteobacterium ancestor of mitochondria into an ancestor of modern eukaryotes most likely produced initially only minor phenotypic variations in the engulfing host. It's only the gradual accumulation of many (naturally selected) variations that transformed progressively the proto-eukaryote into the last common ancestor of modern eukaryotes. This evolutionary process was probably not cataclysmic but more likely take some time since, for instance, 19 eukaryotic specific proteins were added to the ancestral alpha-proteobacterium ribosome (and one bacterial protein lost) between the initial endosymbiosis at the origin of the mitochondrion and the diversification of present-day eukaryotic supergroups (Desmond et al., 2011). **T**here are also many examples of drastic genome reduction in modern endosymbionts (e.g., Rickettsiae) that can be analyzed as the gradual accumulation of gene loss (variation), starting from an ancestor that was a free-living organisms and whose endosymbiotic descendants were gradually selected in a succession of discrete genome reductions steps (Andersson and Andersson, 1999; Renesto et al., 2005).

We should retain from Darwin's gradualist and uniformitarianist views that the basic mechanism explaining descent with modification (variation and selection) have been operating all along life history and, for instance, should serve as framework for origin of life scenarios (Chen et al., 2004). More generally, Darwin adopted the view of those geologists who realize that one should “*explain past changes to the earth in terms of mechanisms that could be studied in the present*” (Penny, 2011). It is essential to preserve this notion of continuity, even if life history went through phases involving very different types of organisms (RNA/peptide cells, RNA/protein cells, RNA/DNA/protein cells, RNA, and DNA viruses). We should imagine scenarios that explain the transition between these forms based on known biological mechanisms, connecting all these forms into convincing evolutionary stories and imagine how one form could have been transformed to another through small or large variations and fixed by selection (either neutral or positive).

## Evolutionary synthesis extended to …. viruses

A recurrent theme in the evolutionary literature is that the “*evolutionary synthesis*” proposed in the middle of the last century should be completed or replaced by an “*extended evolutionary synthesis*” including in particular recent data obtained by studying metazoan development. The origin and evolution of multicellular metazoa is indeed an important aspect of life history, especially from a human perspective. However, macrobes concerned by these processes only represent a tiny fraction of the biosphere (Forterre, 2008). In my opinion, an updated evolutionary synthesis should *in priority* focus on including viruses in the history of life (Brüssow, 2009).

All cellular organisms from the three domains are infected by a plethora of viruses that co-evolved with their hosts and have dramatically altered their history (Prangishvili et al., 2006; Brüssow, 2009; Forterre and Prangishvili, 2009a). However, in the recent excellent textbook for students “*Evolution*” that exposes in parallel molecular biology and evolutionary biology (Barton et al., 2007) viruses are covered in 12 out of 782 pages! This is because viruses have been considered by most biologists to be simple by-products of cellular evolution. Hopefully, this situation is changing (Claverie, 2006; Forterre, 2006b; Brüssow, 2009; Forterre and Prangishvili, 2009b; Villarreal and Witzany, 2010). The qualitative and qualitative importance of viruses in all environments (from the Ocean to the human gut) has been recently realized, thanks to the development of viral ecology and to systematic studies of “viromes” (Rohwer and Thurber, 2009; Kristensen et al., 2010; Rohwer and Youle, 2011). Genomic studies have shown that cellular genomes are full of viral sequences or sequences evolutionary related to viral ones (probably derived from viruses) (Cortez et al., 2009; Feschotte and Clement, 2012). The study of viruses of microbes has made tremendous advances, as testified by the first international meeting devoted to them at the Institut Pasteur in 2010 (Koonin, 2010) and by the recent creation of the international society for the study of viruses of microbes. Viruses have been known for a long time as vehicles of cellular genes. However, viruses are first of all cradles of new genes (selected in viral genomes) and thus providers of new functions. The analysis of viromes has indeed revealed that viruses are the most important source of new genetic information in the biosphere (Rohwer and Youle, 2011 and references therein). This information is directly created during replication/recombination of viral genomes by gene duplication, recombination, insertion, frameshift, gene overlapping, retrotranscription, and so.

I recently introduced the concept of virocell (the infected cell producing virion and no more capable of classical cell division) to emphasize the intracellular creative phase of the virus life cycle (Forterre, 2010a, 2011b, 2012). Indeed, many evolutionists have difficulties to recognize the existence of *bona fide* viral genes. They reason as if all genes present in viral genome should have first originated in either archaeal, bacterial or eukaryotic genomes, before being transferred to viruses. Also, once a viral gene is integrated into a cellular genome, it is considered as a *bona fide* cellular gene in phylogenomic analyses, on the same footing as those inherited from cellular ancestors. For instance, genes present in “prophages” are often confused with “bacterial genes” in phylogenomic or metagenomic analyses. This has important consequences. In particular, introduction of foreign DNA coming from another cellular lineage (by transformation, conjugation or transduction) (true HGT) is not distinguished from introduction of foreign DNA via integration of viral DNA. However, these two types of HGT are completely different. In the first one (true HGT), viruses are not involved, or else eventually play the role of vehicles for cellular gene exchange (transduction), whereas in the second, they provide new genetic material of viral origin to the recipient cell. A prerequisite to understand so-called webs of life (recognizing underlying trees) would be to distinguish between these two types of HGT.

**Figure 2:**
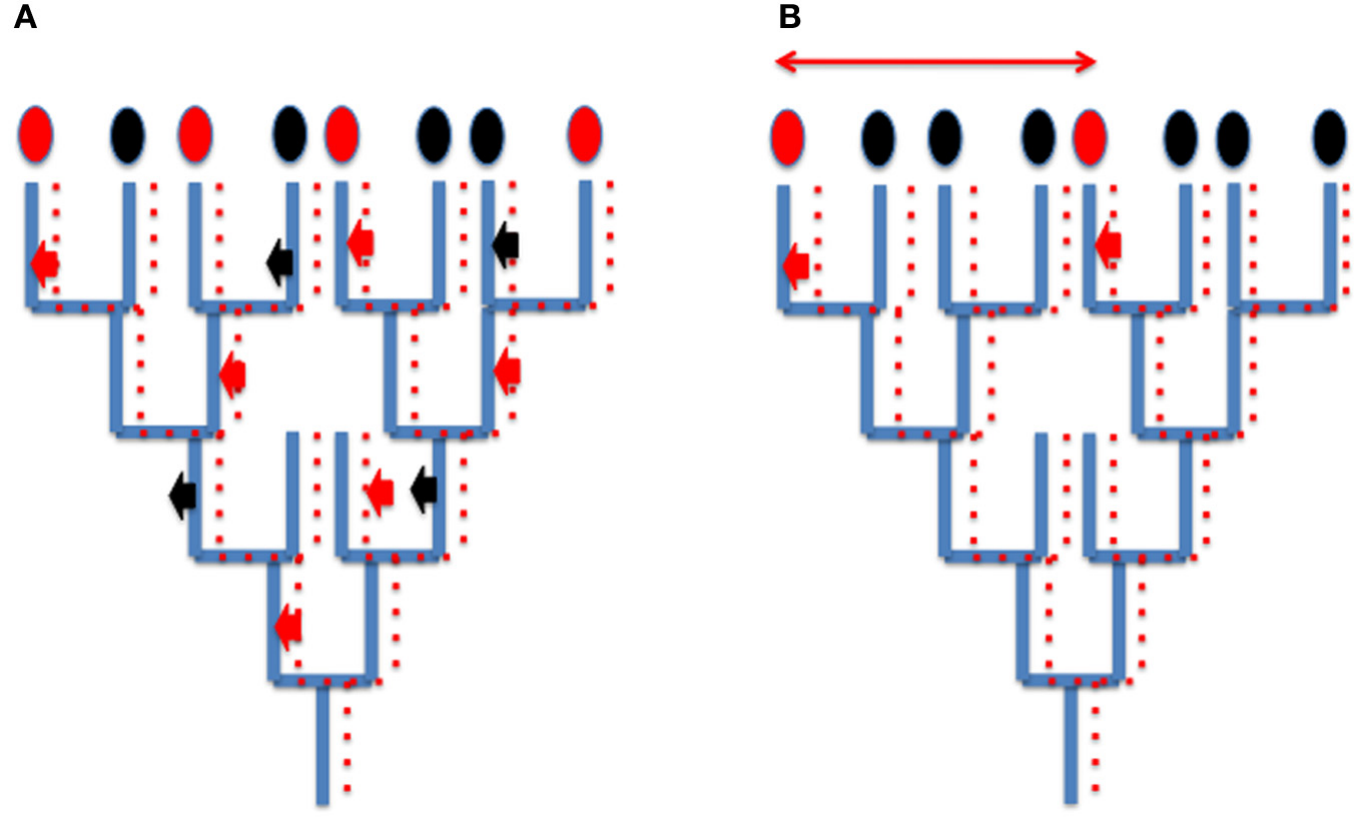
**How integration of viruses or related elements can confuse phylogenetic analyses? (A)** Patchy phylogenetic distribution of viral genes in cellular genomes. A tree of organisms (blue lines) and a co-evolving viral (plasmid) lineages (dotted red lines). A viral (plasmid) gene is sometimes integrated (red arrow) sometimes loss (black arrows) from cellular genomes. The encoded viral proteins will appear as characters present (red ovals) or absent (black ovals) in cellular proteomes. Their use in whole genome tree construction will be misleading, grouping artificially organisms with common integrated viral (plasmid) genes. **(B)** Independent integration of viral genes encoding homologous proteins (small thick red arrows) mimicking horizontal gene transfer (thin red arrow) between two species.

Since viruses and derived elements (plasmids, transposons and retrotransposons) mainly co-evolved with their hosts, HGT corresponding to viral integration does not usually blur the global phylogenetic signal present in cellular genomes (for cases studies, see Krupovic et al., 2010a,b; Soler et al., 2010) although they can produce a patchy distribution of characters that is difficult to interpret (Figure 2A). However, in some case, different viruses can introduce independently homologous viral proteins in different cellular lineages, a situation which will be usually interpreted wrongly as a real HGT between cells (Figure 2B). For instance, since the genomes of head and tailed viruses (Caudovirales) of Archaea and Bacteria sometimes encode homologous proteins (Krupovic et al., 2010a,b), a bacterial-like gene present in an archaeal genome might not testify for the transfer of a bacterial gene into this archaeon, but to the integration into the genome of this archaeon of an archaeovirus encoding a protein homologous to a protein encoded by a related bacteriovirus integrated into a bacterial genome. These confusing effects of integrated viral genes into cellular genomes should not be underestimated, considering that viral genes and evolutionary related elements (plasmids) represent a significant proportion of genomes in the three domains of life (Cortez et al., 2009; Feschotte and Clement, 2012 and references therein).

Viral integration can also mimic gene duplication. For instance many genes supposed to be paralogues (having originated by gene duplication in cellular genomes) might be homologous viral genes that have been introduced several times independently in the same cellular genome (for a case study, see the multiple integration of viral/plasmidic MCM helicases in Methanococcales, Krupovic et al., 2010a,b). Hence, it is unclear if multiple RNA polymerases, DNA polymerases, or MCM subunits in modern eukaryotic genomes originated by gene duplications (the common view) or multiple integrations of viral proteins (Forterre, 2006a).

I will argue here that confusion between cellular and viral genes partly explain the difficulties that many molecular evolutionists have to understand that the “web component” of gene trees (especially microbial ones), does not challenge Darwin but challenges the traditional view that confuse genes of viral and cellular origin.

## Viruses as mediators of biological evolution

Molecular biologists have now shown that viruses and related elements have played a major role in the origin of variations. Their integration into cellular genomes can inactivate cellular genes or promote various forms of genome recombination. Besides, when a viral genome becomes inserted into a cellular genome in regulatory regions, it can promote either activation or inactivation of neighboring genes, modifying the pattern of gene expression (de Parseval and Heidmann, 2005; Feschotte and Clement, 2012). These modifications can be drastic, especially if they touch genes controlling complex regulatory networks. Beside integration, viruses can live in symbiosis (carrier state) with cellular organisms (Ryan, 2007; Villarreal, 2007) providing another major route for the creation of diversity.

Importantly, the integration of viral genomes or the presence of viral symbionts brings at once new genes (hence, possibly new functions) into the cell. Many viral proteins encoded by these genes have been previously selected to interact with cellular proteins and manipulate cellular functions for the virus benefit. These proteins can now be recruited by the cells for their own purpose (exaptation) and help the cells to adapt to viruses but also to many other aspects of their environment (for instance when a bacteria recruited viral toxins to fight their eukaryotic predators). Since viral genomes replicate more often and are quantitatively more abundant than cellular genomes, it is possible that *in fine*, most cellular proteins originated first in the viral world (more precisely in virocells, see Forterre, 2010a, 2011b, 2012) and were only transferred later on into cellular lineages. One can conclude from all these considerations that interaction between viruses and cells has been probably (and still is) a (the) major source of variation (and novelties) in life history.

Darwin wondered about the multiplicative power of life, he would have been fascinated by the incredible multiplicative power of viruses. The huge number of infectious viral particles present in the biosphere has imposed a dramatic selection pressure (natural selection in grand scale) to natural populations all along life history. This has now been clearly established for modern marine viruses that fundamentally “*manipulate*” their environment, controlling the structure of microbial populations (Rohwer and Thurber, 2009 and references therein). Similarly, retroviruses and derived genetic elements have imposed a dramatic selection pressure all along eukaryotic evolution, as testified by the huge number of endogenous retroviruses and derived elements now integrated into animals or plant genomes (Brosius, 2003; de Parseval and Heidmann, 2005; Feschotte and Clement, 2012). In fact, the major problem faced by any cellular population is how to adapt to their viral environment.

Considering the impact of viruses on both selection and variation, the two pillars of Darwin's core idea, the conflict between viruses and cells has been (and still is) probably the main engine of biological evolution (Forterre and Prangishvili, 2009b). In particular, the arm race between viruses and cells could partly explain the apparent tendency of life evolution toward complexity. This arm race has been probably a major source of novelties in the living world, much like arm races between tribes, cities and states have been a major factor of novelties in human history. More generally, the existence of parasites has been certainly a constant of life history (in agreement with uniformitarianism). As theoretically shown by Penny and co-workers in the case of the conflict between phagotrophs and their preys, “*there was no garden of Eden*” at the time of LUCA or before (De Nooijer et al., 2009), i.e., a “communal” word without parasites has never existed. The conflict between proto-viruses and RNA cells and later on between viruses and cells was probably a major evolutionary force at several critical steps in the history of life. This possibility has been explored to explain for instance the origin of DNA (Forterre, 2002) the origin of cell wall (Jalasvuori and Bamford, 2008), or else the emergence of unique eukaryotic features, such as the nuclear membrane, the telomere or else the odd mRNA capping structures (Forterre, 2011a and references therein).

It has been often claimed by anti-Darwinists, that the simple process of random mutations is not powerful enough to have produced the complexity and diversity of modern life forms. This is probably partly because they never consider in their reasoning the role that viruses have played in shaping cellular evolution. Unfortunately, Darwin was unaware of the existence of viruses, he would have been thrilled by the powerful tools for biological evolution hidden before our eyes, all these myriad of tiny “Darwinists” working days and nights to slowly but constantly change the face of the planet by promoting variation and selection.

## Conclusion

At the dawn of the XXI century, some biologists apparently dream to bypass Darwin. For me, this is hopeless, except if we reduce Darwin to some *ad hoc* version of « Darwinism » or if we consider Darwin as one of our contemporaries, and not a scientist of the XIX century. With the dyad variation/selection, Darwin has provided us with key concepts that are necessary and sufficient to understand the logic of evolution, a goldmine that is still open. All the striking discoveries made in biology during the last 150 years have been extensions of these concepts and recent discoveries in microbial evolution and post-genomic studies are not different. We cannot bypass Darwin, but we can go beyond Darwin by the continuous exploration of the biosphere and the many particular mechanisms of life evolution. These mechanisms are much more diverse and sometimes complex than those imagined at the turn of the last century and new unexpected mechanisms certainly remained to be discovered. Of course, if we plan to reconstruct the history of life itself, especially those of ancient life, we are face to immense difficulties. However, we should not try to escape these difficulties by replacing trees by networks. In the meantime, we should go back to the fields to complete our inventory of microbes and their viruses, and be grateful to Darwin, who teaches us to look nature with open eyes beyond the veil of ideologies.

### Conflict of interest statement

The author declares that the research was conducted in the absence of any commercial or financial relationships that could be construed as a potential conflict of interest.
